# Oligodendrocyte dysfunction in neurodegenerative diseases: pathological features, underlying mechanisms and therapeutic targeting

**DOI:** 10.3389/fnagi.2026.1843643

**Published:** 2026-05-15

**Authors:** Zizhen Liu, Jingjing Liu, Junjing Liang, Chang Meng, Qinglu Wang, Ying Luo, Haibin Zhang

**Affiliations:** 1 College of Basic Medicine, Qilu Medical University, Zibo, Shandong, China; 2 College of Sport and Health, Shandong Sport University, Jinan, China; 3 Department of Clinical Laboratory, Zibo Central Hospital, Zibo, China

**Keywords:** metabolic disorder, myelin damage, neurodegenerative diseases, oligodendrocytes, targeted therapy

## Abstract

Oligodendrocyte lineage cells, consisting of mature oligodendrocytes (mOLs) and their progenitors (OPCs), sustain myelination and support axonal metabolic integrity in the mammalian central nervous system. In neurodegenerative disorders, functional deficits spanning impaired mOL homeostasis and dysregulated OPC activation are no longer regarded as passive secondary outcomes of neuronal injury. Instead, emerging clinical and preclinical data demonstrate that oligodendroglial dysfunction actively fuels disease progression. Notably, most human evidence remains correlative, with few definitive proofs that OL pathology initiates neurodegeneration, indicating lineage malfunction predominantly exacerbates, rather than triggers, disease onset across Alzheimer's, Parkinson's, and Huntington's diseases. In this review, we synthesize disease-specific oligodendrocyte pathological signatures and context-dependent cellular responses, focusing on underrecognized OL-intrinsic pathogenic mechanisms: endogenous Aβ production, aberrant protein aggregation, disrupted cholesterol turnover, and excessive neuroinflammatory amplification. We further establish a unified mechanistic model to explain the widespread heterogeneity of white matter pathology across distinct neurodegenerative contexts. We detail four core interconnected pathways whereby defective OL lineage function drives tissue deterioration: myelin loss and progressive axonal degeneration, disrupted neuroimmune homeostasis, cytotoxicity from aggregated pathological proteins, and dysregulated metabolic signaling. To resolve persistent conceptual confusion in the field, we strictly distinguish cell-autonomous primary oligodendroglial lesions, secondary reactive changes following neuronal damage, and non-specific white matter remodeling. We also address critical translational barriers stemming from well-documented phenotypic discrepancies between animal models and human patient brains. Moreover, we consolidate current OL-targeted therapeutic strategies, including myelin restoration, immunomodulatory intervention, metabolic reprogramming, and gene-targeted therapy, highlighting the clinical bottlenecks of single-target regimens and the superior translational prospects of multi-target combinatorial strategies. We conclude by outlining key unresolved challenges and future research avenues, covering OL subtype identification, intercellular signaling crosstalk characterization, humanized model optimization, and precision delivery technique innovation. Collectively, this review refines our understanding of context-dependent oligodendrocyte biofunctions in neurodegeneration, clarifies the origin and consequence of white matter lesions, and offers actionable mechanistic and theoretical support for developing novel glia-based clinical therapies.

## 1 Introduction

Oligodendrocytes (OLs) are the primary glial cells of the central nervous system. Oligodendrocyte lineage cells consist of mature oligodendrocytes (mOLs) and oligodendrocyte precursor cells (OPCs). mOLs primarily form myelin sheaths and provide axonal metabolic support, OPCs maintain proliferative and differentiation potential for myelin regeneration. Their primary functions include forming myelin sheaths to ensure the transmission of neural signals and providing metabolic and nutritional support to axons; they are essential for maintaining the homeostasis of central nervous system function (Looser et al., 2024). Recent advances in technologies such as single-cell omics and gene editing have further revealed that mature OLs and OPCs participate in the regulation of neural circuit homeostasis by modulating synaptic pruning and balancing neurotransmitter levels, challenging the traditional view that they are merely “neural support cells” (Temple, 2023; Zou et al., 2026).

Neurodegenerative diseases such as Alzheimer's disease, Parkinson's disease, and Huntington's disease are characterized by progressive neuronal degeneration. While previous studies have primarily focused on pathological changes in neurons, the role of OL dysfunction in these diseases has long been overlooked. Recent studies have confirmed that OL dysfunction is not merely a secondary manifestation of disease, but may act as a critical contributor associated with disease progression rather than initiation, with robust causal evidence only in preclinical models and limited validation in human patients (Festa et al., 2024; Ziar et al., 2025; Zou et al., 2023). OLs and OPCs exhibit specific pathological changes and context-dependent responses across different diseases, offering a new perspective for research into disease mechanisms and the development of therapeutic targets.

However, many critical scientific questions remain unresolved in this field: the molecular characteristics, functions and cell-autonomous properties of disease-specific OL subtypes have not yet been clarified (Mathys et al., 2023); the mechanisms underlying the cross-talk between OL lineage cells and other neuronal cells remain unclear; and while the clinical translation of existing single-target therapies is limited, the synergistic mechanisms of multi-target combination therapies still need to be explored. We also strictly distinguish between primary cell-autonomous OL pathology, secondary responses to neuronal degeneration, and disease-associated white matter changes.

Based on this, this review focuses on the functional abnormalities of OLs and OPCs, systematically summarizes their disease-specific pathological manifestations and broadly shared stress responses in major neurodegenerative diseases, and uses a unified mechanistic model to explain the specificity and commonality of OL lesions in different diseases, and analyzes the cross-regulatory relationships among core mechanisms by distinguishing cell-intrinsic dysfunction and intercellular crosstalk disorders such as myelin damage and immune-inflammatory imbalance; It comprehensively summarizes the progress of therapeutic research targeting the oligodendrocyte lineage, analyzing the advantages and limitations of various strategies; and, by integrating cutting-edge technologies, outlines future key research directions. The aim is to provide new perspectives for elucidating the hierarchical and context-dependent mechanisms of white matter damage in these diseases and to lay a theoretical foundation for the development and clinical translation of OL-targeted therapeutic strategies.

## 2 Pathological manifestations of OLs in major neurodegenerative diseases

### 2.1 Alzheimer's disease (AD)

#### 2.1.1 Abnormal proliferation of OLs and enhanced Aβ production capacity

Recent studies indicate that, as an emerging and provocative concept requiring further validation, mOLs may represent an important non-neuronal source of Aβ in AD, with their abnormal proliferation and functional remodeling further accelerating the pathological process (Sasmita et al., 2024). Specifically, the number of OLs in layers 5 and 6 of the prefrontal cortex was significantly increased in AD patients, whereas no similar changes were observed in layers 2 and 3, suggesting that the pathological changes are region-specific (Mathys et al., 2023). In addition, approximately 80% of mature OLs express both APP and BACE1, conferring upon them the full capacity to generate Aβ; furthermore, the number of such cells is significantly higher in AD patients than in controls, indicating that OLs are one of the major sources of Aβ in the brain (Simons et al., 2024).

Single-cell RNA sequencing analysis revealed that OLs highly express multiple genes associated with Aβ production, including APP, BACE1, PSEN1, and NCSTN, particularly BACE1 (Simons et al., 2024). Experimental findings further confirmed that OLs derived from AD patient-derived human induced pluripotent stem cells (iPSCs) not only produce large amounts of Aβbut also exhibit a significantly higher Aβ42/Aβ40 ratio than their isogenic neuronal counterparts and show an increased propensity to form neurotoxic soluble aggregates (20–200 nm), with their quantities far exceeding those produced by neurons (Table 1; Simons et al., 2024).

**Table 1:** Summary of oligodendroglial pathological phenotypes, key molecules/pathways and evidence strength in major neurodegenerative diseases.

Disease	Oligodendroglial pathological phenotype	Key implicated molecules/pathways	Evidence strength	References
Alzheimer's disease (AD)	OL abnormal proliferation; OL-derived Aβ production; tau aggregation in OLs; cholesterol metabolism disorder; myelin synthesis defect	APP, BACE1, PSEN1, NCSTN, APOE4, tau, BIN1	Human correlative evidence; mouse model causal evidence; human iPSC evidence	Blanchard et al., 2022; Mathys et al., 2023; Oatman et al., 2026; Simons et al., 2024
Parkinson's disease (PD)	Myelin structure loosening; α-syn aggregation and transmission; neuroinflammatory amplification	PLP1, MBP, GPR37, PSAP, IL-6, α-syn	Mouse model causal evidence; human correlative evidence; *in vitro* evidence	Ma et al., 2025; Wihan et al., 2025; Zhang et al., 2025
Huntington's disease (HD)	OPC maturation impairment; myelin gene downregulation; axonal metabolic support defect; mild tau phosphorylation	mHTT, OLIG2, SOX10, BDNF, GDNF	Mouse model causal evidence; *in vitro* evidence; human correlative evidence	Heinzmann et al., 2026; Li X. et al., 2025; Lim et al., 2022

Animal model studies provide direct causal evidence in preclinical systems that OL-derived Aβ contributes to neuronal dysfunction during the early symptomatic stages of AD; extrapolation to human disease initiation requires further clinical validation. In APP NL-GF knock-in mice, specific knockout of BACE1 in OLs reduces the number of Aβ plaques in the brain by 25% and significantly decreases plaque area in vulnerable regions such as the hippocampal CA1 region and the corpus callosum (Sasmita et al., 2024). Further studies revealed that soluble Aβ aggregates secreted by OLs can directly induce neuronal hyperactivity; injecting conditioned medium containing these aggregates into the retrosplenial cortex of wild-type mice rapidly increased neuronal firing rates, whereas depletion of Aβ or pretreatment with a BACE1 inhibitor completely abolished this effect (Huang et al., 2025; Simons et al., 2024).

#### 2.1.2 The effects of tau protein deposition on OLs function

Tau protein deposits may impair OL function through direct and indirect pathways, with distinct processes that are partially demonstrated or inferential. Directly, mature OLs can internalize neuron-derived tau protein, leading to intracellular aggregation, mitochondrial dysfunction, and apoptosis. In the hippocampus of AD patients, neuronal loss around tau-positive oligodendrocytes is more pronounced (Ziar et al., 2025). Animal model studies have further confirmed that the specific expression of mutant tau in OLs can lead to impaired axonal transport, abnormal myelin structure, and cell-autonomous damage (Majumder and Dutta, 2024).

Indirectly, tau pathology affects the synaptic support and myelin repair functions of oligodendrocytes. In the AD brain, tau oligomers can be recognized by OLs, which promote excessive synaptic phagocytosis via the C1q-dependent complement pathway; knocking out C1q alleviates synaptic loss in tau transgenic mice (Chen et al., 2023). In addition, tau overexpression impairs the repair capacity of oligodendrocyte precursor cells (OPCs): although differentiation activity is transiently enhanced, the stability of newly formed myelin is reduced, making them prone to secondary degeneration, which leads to impaired regeneration (Ziar et al., 2025). Additionally, the AD-associated gene BIN1 interacts with tau in mature OLs, further promoting tau phosphorylation and aggregation, thereby exacerbating functional impairment (Oatman et al., 2026).

#### 2.1.3 Cholesterol metabolic disorders and myelin synthesis defects

Dysregulation of cholesterol metabolism is a key factor in the impairment of myelin synthesis in Alzheimer's disease and is closely associated with the major risk gene APOE4 (Zou et al., 2026). APOE4 is expressed in OLs and disrupts cholesterol homeostasis: on the one hand, abnormal expression of cholesterol transporters and synthases leads to intracellular cholesterol accumulation and impaired efflux (Blanchard et al., 2022); experiments have confirmed that cholesterol efflux efficiency in APOE4-expressing cells is approximately 40% lower than in wild-type cells. On the other hand, the proportion of cholesterol in myelin lipids decreases, directly affecting the stability and integrity of the myelin structure (Litvinchuk et al., 2024). These findings are well-supported in preclinical models, while translation to human disease requires further validation.

Animal model studies have shown that APOE4 knock-in mice exhibit significant myelin thinning and structural disorganization in old age, whereas specifically enhancing the cholesterol transport capacity of oligodendrocytes not only restores myelin thickness but also reduces Aβ deposition (Al-Amin et al., 2025). Mechanistically, dysregulation of cholesterol not only directly affects myelination but also exacerbates Aβ accumulation by modulating γ-secretase activity and impairing microglial phagocytosis (Huang et al., 2025; Ziar et al., 2025).

### 2.2 Parkinson's disease (PD)

#### 2.2.1 Dysfunction of oligodendrocytes associated with dopaminergic neurons in the midbrain

**Figure 1:**
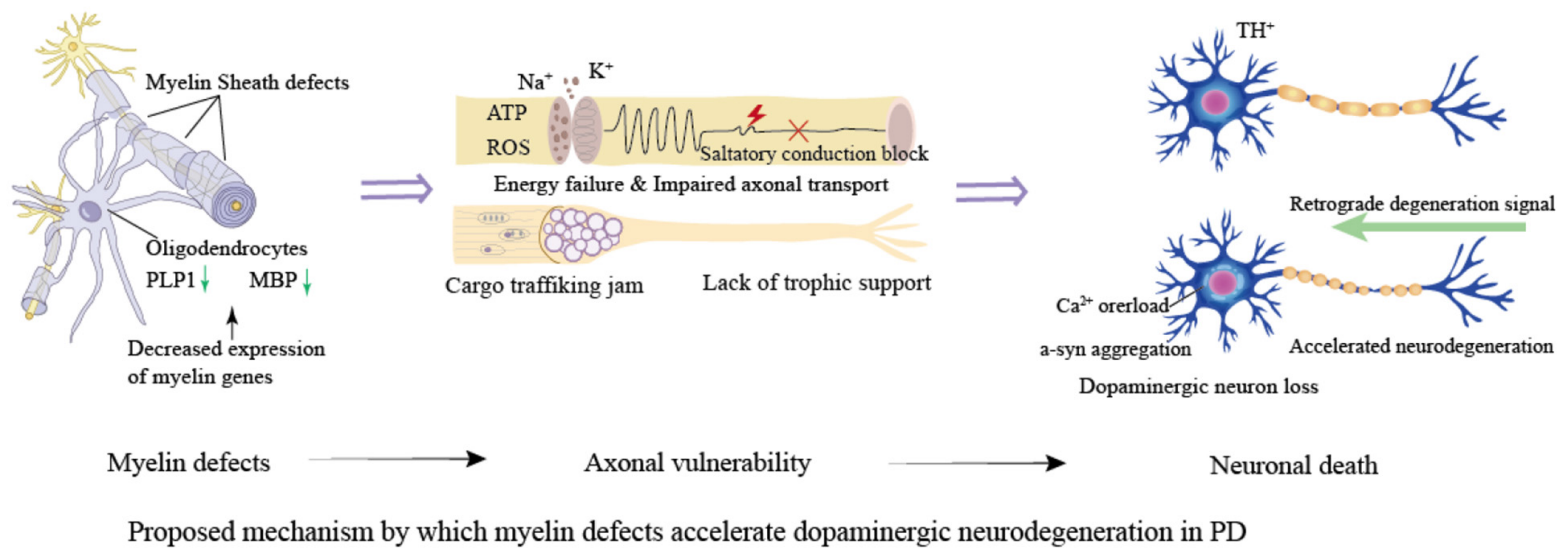
In PD models, downregulation of myelin synthesis genes (such as PLP1 and MBP) leads to loose myelin structure and reduced myelin thickness. This defect compromises axonal insulation and saltatory conduction, resulting in impaired transmission and energy metabolism disorders. The resulting failure of axonal transport deprives distal dopaminergic terminals of nutritional support, triggering “backward death” signals, which ultimately accelerate the degeneration of dopaminergic neurons, creating a vicious cycle of neuronal damage.

In PD, functional abnormalities in mature OLs associated with midbrain dopaminergic neurons represent a key pathological mechanism. These changes largely represent mOL responses to dopaminergic neuron injury, while primary mOL autonomous pathology in PD remains to be fully defined. Under normal conditions, mature OLs support neurons by maintaining myelin and the metabolic microenvironment; their dysfunction exacerbates neuronal damage (Li Y. et al., 2025). In PD models, downregulation of myelin synthesis genes (such as PLP1 and MBP) leads to loose myelin structure, reduced myelin thickness, and impaired axonal conduction, thereby accelerating the degeneration of dopaminergic neurons (Figure 1; Li Y. et al., 2025).

Mechanistically, abnormal upregulation of GPR37 drives the progression of PD. GPR37 is highly expressed in PD models; however, its absence can mitigate neuronal damage and improve motor deficits by maintaining myelin homeostasis and reducing the release of toxic factors (Liu et al., 2025). Meanwhile, OPC transplantation can repair myelin and improve motor deficits, further confirming that dysfunction of OL lineage cells contributes to the pathological process of PD (Dehestani et al., 2024).

#### 2.2.2 Aggregation and propagation of α-synuclein in OL lineage cells

Abnormal aggregation of α-synuclein (α-syn) in mature OLs is capable of spreading from cell to cell. In patients with PD, the level of phosphorylated α-syn deposits in OLs within the substantia nigra pars compacta and striatum correlates positively with disease severity (Wihan et al., 2025).

In A53T transgenic mice, α-syn aggregation within mature OLs inhibits their differentiation and maturation, reduces the number of OLIG2^+^ mature OLs, downregulates myelin gene expression, and exacerbates cellular dysfunction by inhibiting the autophagy-lysosomal pathway (Bae et al., 2026). Pathological α-syn can also be transmitted from OLs to neighboring neurons and glial cells via gap junctions or extracellular vesicles. GPR37 deficiency significantly reduces α-syn aggregation, suggesting it as a potential intervention target (Liu et al., 2025).

#### 2.2.3 The neuroinflammatory amplification effect mediated by mature OLs

Neuroinflammation plays a critical role in the progression of PD, and mature OLs amplify the inflammatory response via the PSAP–GPR37–IL-6 axis. In PD models, increased levels of phosphatidylserine-activated protein (PSAP) secreted by dopaminergic neurons bind to GPR37 on the surface of OLs, thereby inducing the expression and secretion of IL-6 via the Gαi/MEK pathway (Ma et al., 2025). IL-6 directly damages neurons and activates microglia, leading to the release of pro-inflammatory factors such as TNF-α and IL-1β, which form an inflammatory feedback loop that exacerbates neuronal damage (Wang et al., 2026).

Elevated levels of PSAP and IL-6 in the cerebrospinal fluid and brain tissue of PD patients are associated with disease progression. Conditional knockout of mature OL GPR37 or PSAP reduces IL-6 levels, inhibits glial cell activation, and alleviates neuronal damage and motor deficits. IL-6 secreted by OLs also promotes iron accumulation in neurons via trans-signaling pathways, thereby amplifying neurotoxicity (Ma et al., 2025). In summary, mature OLs mediate the amplification of neuroinflammatory cascades via the PSAP–GPR37–IL-6 axis, acting as an important amplifier of PD pathological progression and a potential therapeutic target; this has been demonstrated in models, with human correlative evidence (Table 1).

### 2.3 Huntington's disease (HD)

HD is an autosomal dominant neurodegenerative disorder caused by an abnormal expansion of CAG repeats in the HTT gene (Heinzmann et al., 2026). Studies have shown that dysfunction of oligodendrocyte lineage cells plays a significant role in HD progression. The core pathological features center on mutant huntingtin (mHTT)-dependent transcriptional dysregulation, impaired OPC maturation, downregulated myelin gene expression and defective axonal metabolic support. Tau phosphorylation and aggregation are secondary concomitant features rather than core pathological events in HD oligodendroglial dysfunction (Alpaugh et al., 2022). OL dysfunction in HD is mainly a secondary reactive change induced by mHTT, and causal evidence supporting OL dysfunction as an initiator of HD is extremely limited.

Mutant huntingtin (mHTT) disrupts transcriptional programs in **OPCs and mature OLs**, inhibiting the expression of myelin-related genes such as OLIG2 and SOX10, and compromising myelin integrity. mHTT directly inhibits OPC differentiation into mature OLs, leading to reduced myelin production and unstable myelin sheaths, further affecting axonal structural and functional stability (Li X. et al., 2025). The ability of **mature OLs** to secrete neurotrophic factors (such as BDNF and GDNF) is reduced, weakening **metabolic and trophic support** for neurons (Table 1).

As a secondary event, mHTT interacts with tau to promote mild tau phosphorylation and aggregation, which further impairs protein clearance but is not a core driver of OL dysfunction in HD. Functional experiments indicate that transplanting normal OPCs can improve motor and cognitive deficits in HD model mice by promoting myelin repair and neurotrophic factor secretion; inhibiting tau phosphorylation also alleviates myelin damage and neuronal degeneration (Li J. et al., 2025; Li W. et al., 2025).

## 3 The core mechanisms of OLs in neurodegenerative diseases

### 3.1 Myelin damage and axonal degeneration

#### 3.1.1 Lack of axonal metabolic support and insufficient energy supply

Mature OLs form tight connections with axons via myelin sheaths, providing metabolic support such as lactate shuttling and the delivery of neurotrophic factors. In neurodegenerative diseases, impaired mature OL function disrupts axonal metabolic support, acting as a core mechanism that accelerates axonal degeneration; in some cases, this is secondary to initial neuronal injury (Looser et al., 2024). OLs supply energy to axons via the monocarboxylate transporter (MCT1). In AD, decreased MCT1 expression in OLs reduces lactate uptake by axons and leads to insufficient ATP production, resulting in functional impairment (Lee et al., 2012). The APOE4 genotype disrupts cholesterol metabolism in OLs, compromising myelin maintenance and axonal stability, thereby further exacerbating the metabolic crisis (Blanchard et al., 2022). Improving the metabolic function of OLs can effectively alleviate axonal degeneration (Looser et al., 2024).

In the PD model, the loss or dysfunction of mature OLs leads to an axonal energy crisis, manifested by mitochondrial dysfunction, reduced oxidative phosphorylation efficiency, depletion of glycogen stores (Zhang et al., 2025), and decreased secretion of neurotrophic factors (such as BDNF). These factors collectively result in a lack of metabolic support and insufficient energy supply to the axon, ultimately leading to axonal degeneration and neuronal death.

#### 3.1.2 A chronic inflammatory microenvironment caused by impaired clearance of myelin debris

**Figure 2:**
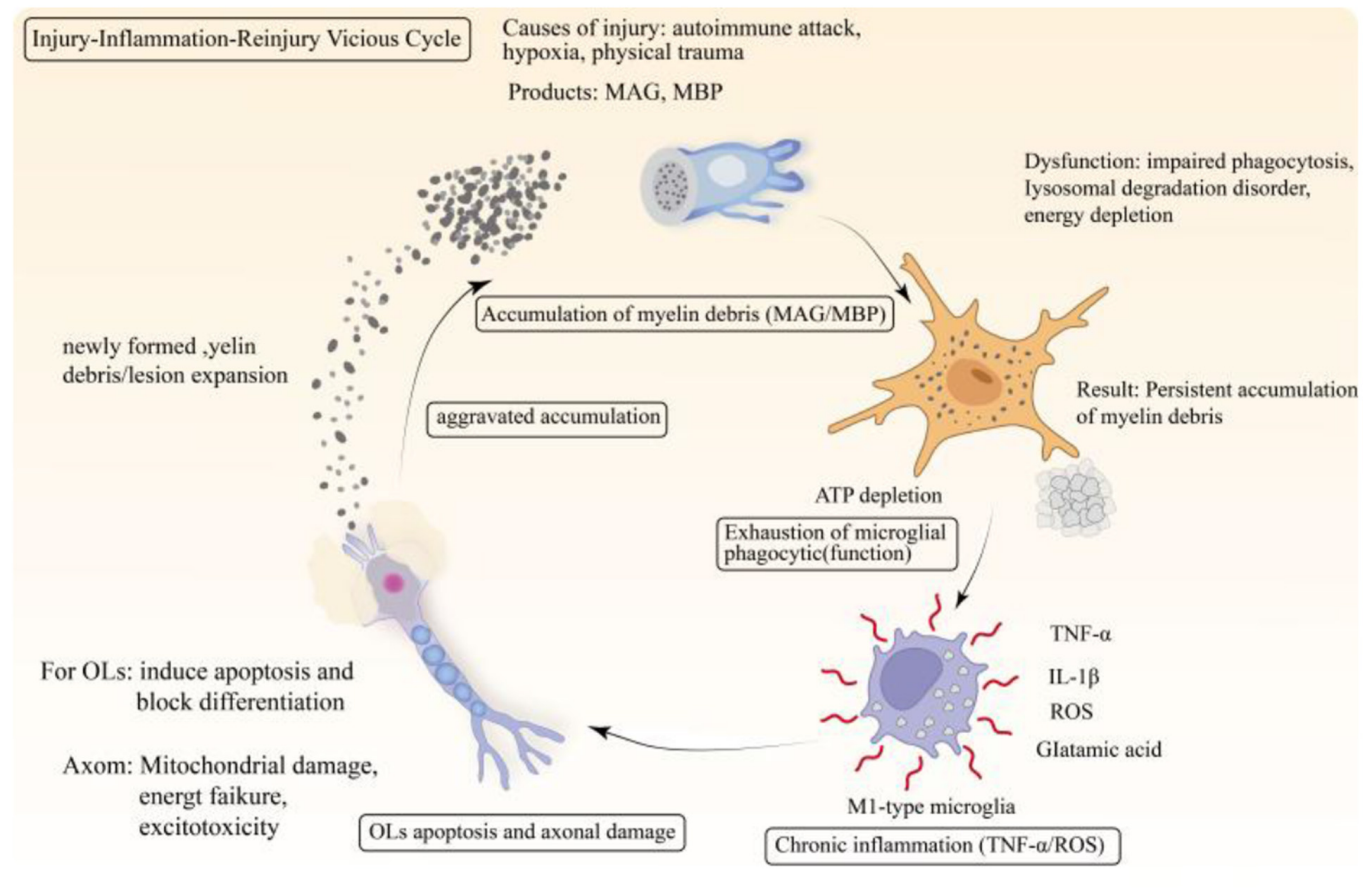
Myelin debris clearance failure drives a “injury-inflammation-reinjury” vicious cycle: when the clearance of myelin fragments is impaired, the accumulated fragments continuously activate microglia, prompting them to release inflammatory factors, reactive oxygen species, and glutamate. These mediators induce apoptosis in OLs while also damaging axons, leading to the generation of new myelin and axonal fragments. The newly formed fragments further increase the clearance burden and amplify the inflammatory response, thereby creating a self-perpetuating vicious cycle of “injury → inflammation → re-injury” that drives the chronic progression of the disease.

Impaired clearance of myelin debris can trigger chronic inflammation, which continuously attacks OL lineage cells and axons, creating a vicious cycle of “injury-inflammation-re-injury” (Figure 2). This is one of the core mechanisms underlying the progression of neurodegenerative diseases. Under physiological conditions, microglia and astrocytes are responsible for clearing myelin debris to support repair. In neurodegenerative diseases, pathological proteins such as Aβ inhibit the phagocytic function of microglia, leading to impaired clearance of myelin debris (Njoku and Kanmogne, 2025). Accumulated debris can activate microglia to release pro-inflammatory factors and induce astrocytes to adopt a neurotoxic phenotype, jointly creating a chronic inflammatory microenvironment that ultimately exacerbates OL damage and axonal degeneration (Figure 2; Kedzia et al., 2026).

In neurodegenerative diseases, impaired clearance of myelin debris is a key factor in the development of a chronic inflammatory microenvironment. Accumulated myelin debris directly damages OLs by activating the complement pathway to generate C5b-9 complexes; simultaneously, the myelin proteins released from this debris can be recognized by antigen-presenting cells, inducing persistent autoimmune attacks that hinder myelin repair. Promoting debris clearance can effectively reduce inflammation and improve the repair process (Li C. et al., 2025). In PD, α-synuclein aggregates bind to myelin debris, synergistically activating microglia, exacerbating the inflammatory response, and leading to dopaminergic neuron damage (Tian et al., 2026). In HD, the mutant huntingtin protein promotes chronic inflammation by inhibiting OLs autophagy and reducing the degradation of myelin debris (Li X. et al., 2025). In summary, impaired clearance of myelin debris is a common mechanism underlying the chronic inflammatory microenvironment in various neurodegenerative diseases.

### 3.2 Imbalance in immune and inflammatory regulation

#### 3.2.1 MHC molecule expression and antigen presentation in OLs

Under normal conditions, mature OLs express low levels of MHC molecules and have limited antigen-presenting capacity (Catenacci et al., 2025). However, in the inflammatory environment of neurodegenerative diseases, their MHC expression and antigen-presenting function are significantly enhanced, thereby activating autoimmune responses and exacerbating the neuroinflammatory process (Catenacci et al., 2025). In AD, Aβ and tau pathology can induce OLs to abnormally express MHC class I molecules, enabling them to present self-antigens (such as myelin proteins), thereby activating T cells and exacerbating neuroinflammation (Kedia et al., 2024). This process suggests that OLs are involved in the dysregulation of central immune inflammation, which may be associated with disease progression.

#### 3.2.2 Regulation of OLs by cytokine networks

In neurodegenerative diseases, cytokine networks within the microenvironment (including TNF, IFN-γ, and IL-17) directly regulate the survival, differentiation, and function of OPCs and mature OLs through signal transduction, thereby exacerbating pathological damage.

Most cytokine effects are defined in inflammatory or demyelinating models and may differ from chronic low-grade inflammation in bona fide neurodegenerative diseases.

##### 3.2.2.1 Tumor necrosis factor (TNF)

TNF exerts bidirectional immunoregulation on OLs via TNFR1 and TNFR2. On the one hand, soluble TNF binds to TNFR1 to induce apoptosis, while transmembrane TNF promotes cell survival and myelin repair through TNFR2 (Lei and Lin, 2024). Under chronic inflammatory conditions, TNF signaling shifts toward the TNFR1 pathway, exacerbating OL damage and inhibiting the differentiation of their precursor cells, leading to failed myelin repair (Zhang et al., 2024). Furthermore, in AD, TNF can also promote Aβ production by OLs and suppress the expression of myelin-related genes, thereby aggravating myelin damage (Blanchard et al., 2022).

##### 3.2.2.2 Interferon-γ (IFN-γ)

The regulatory effects of IFN-γ on OLs function are concentration- and time-dependent. Sustained exposure to high levels of IFN-γ is a key factor contributing to OLs damage. Under chronic pathological conditions, high concentrations of IFN-γ can directly induce apoptosis and inhibit the differentiation of OPCs and myelination through endoplasmic reticulum stress (Chen et al., 2023). Concurrently, IFN-γ disrupts cholesterol metabolism, reduces myelin stability, and creates a vicious cycle that promotes inflammation (Blanchard et al., 2022). Although low concentrations of IFN-γ exert short-term protective effects, its long-term chronic effects are predominantly detrimental (Lei and Lin, 2024).

##### 3.2.2.3 Interleukin-17 (IL-17)

IL-17 directly inhibits the differentiation of OPCs by activating the NOTCH1 pathway and drives their secretion of chemokines, thereby recruiting immune cells (Wang et al., 2025). Concurrently, IL-17 enhances the pro-inflammatory phenotype of OLs, promotes the release of inflammatory mediators and reactive oxygen species, and exacerbates myelin damage. In AD, IL-17 acts synergistically with Aβ to further impair the repair function of OLs, leading to impaired myelin regeneration (Depp et al., 2023).

In addition, pro-inflammatory factors such as IL-1β and IL-33 inhibit the proliferation and differentiation of OPCs via the p38-MAPK pathway, while anti-inflammatory factors such as IL-4 and TGFβ can partially counteract this effect. This suggests that an imbalance in the cytokine network is a key mechanism regulating the pathological state and reparative function of OLs (Zhang et al., 2024).

### 3.3 Abnormal protein deposition and cytotoxicity

#### 3.3.1 Mechanisms of Aβ, tau, and α-synuclein aggregation within OL lineage cells

In neurodegenerative diseases, various abnormal proteins can accumulate abnormally within OLs; the mechanisms underlying this aggregation are closely related to the proteins' intrinsic properties, cellular uptake and processing abnormalities, and is often secondary to neuronal proteinopathy rather than cell-autonomous initiation. Most mechanistic data are from rodent models, with partial validation in human iPSC-derived OLs and limited *in vivo* human evidence. OLs possess a complete molecular mechanism for Aβ production, exhibiting high expression levels of APP, BACE1, and gamma-secretase components, with some expression levels even exceeding those in neurons (Sasmita et al., 2024). In the brains of AD patients, the number of OLs capable of producing Aβ is significantly increased, directly expanding the pool of Aβ production within the brain (Simons et al., 2024). Furthermore, OLs can actively uptake extracellular Aβ via endocytosis (a process exacerbated by the ApoE4 genotype) while simultaneously inhibiting its intracellular degradation, collectively promoting the aggregation of Aβ within the cells (Konings et al., 2023).

Tau protein aggregates within oligodendrocytes, a process primarily involving intercellular transmission and abnormal intracellular processing. Tau secreted by neurons can be taken up by oligodendrocytes via endocytosis, where it forms aggregates within the cells (Al Kabbani et al., 2025). The expression of mutant tau (such as P301L) directly induces aggregation, while abnormal expression of the risk gene BIN1 exacerbates this process by affecting microtubule stability and other mechanisms (Smith et al., 2025).

OLs form intracellular aggregates by taking up pathological α-synuclein derived from neurons. These aggregates inhibit the autophagy-lysosomal pathway, impede oligodendrocyte differentiation and maturation, and downregulate myelin gene expression. The aggregates can spread to neurons and other glial cells via gap junctions and extracellular vesicles, thereby amplifying pathological damage (Wihan et al., 2025). GPR37 deficiency significantly reduces α-syn aggregation, suggesting that it is a key target regulating uptake or aggregation.

#### 3.3.2 Toxic protein-induced apoptosis and functional failure in OLs

The aggregation of abnormal proteins within OLs can directly induce apoptosis or lead to the failure of their core functions, thereby compromising myelin integrity and the support provided to neurons (Festa et al., 2024). In OLs, Aβ aggregation can directly trigger caspase-dependent apoptotic pathways, resulting in a reduction in cell numbers (Haque et al., 2026). Concurrently, Aβ further impairs the ability to maintain and repair myelin by inhibiting key functions such as cholesterol synthesis and transport, thereby exacerbating cellular dysfunction. Abnormal tau protein aggregation within OLs disrupts microtubule stability and interferes with the transport and synthesis of myelin proteins. Furthermore, tau aggregation can induce oxidative stress and mitochondrial damage, ultimately leading to OLs apoptosis, impaired myelination, and neurological dysfunction (Ferreira et al., 2020).

#### 3.3.3 The impact of misfolded and propagating proteins on the OL population

The misfolding and intercellular transmission of abnormal proteins can trigger a cascade of reactions, leading to functional disruption in OLs and subsequently exacerbating neurodegenerative pathology. The misfolding and transmission of Aβ promote pathological activation of OLs. Aβ produced by these cells exhibits a stronger tendency to aggregate; it has a higher Aβ42-to-Aβ40 ratio compared to neuron-derived Aβ, making it more prone to forming soluble aggregates (Simons et al., 2024). These aggregates activate the pro-inflammatory phenotype of surrounding OLs via paracrine mechanisms, releasing inflammatory cytokines such as IL-6 and TNF-α, which in turn inhibit myelin repair. Under acute demyelination conditions, this process can further propagate through neuroinflammation, leading to more extensive myelin damage.

Misfolded tau protein can spread among OLs, leading to abnormal intracellular deposits. These deposits cause dysfunction in OLs; some cells exhibit impaired repair capacity, while others display excessive inflammatory responses, ultimately disrupting the coordinated function of the cell population (Majumder and Dutta, 2024).

In PD, misfolded α-synuclein can spread to OLs and propagate throughout the population. This directly leads to impaired cellular function, manifested as downregulation of myelin synthesis genes (Wihan et al., 2025). Concurrently, deposited abnormal proteins activate the cells' innate immune response, triggering chronic inflammation at the population level, which ultimately exacerbates cytotoxicity and neuronal damage (Bae et al., 2023).

### 3.4 Metabolic and signaling pathway dysregulation

#### 3.4.1 The pathogenic role of cholesterol and abnormal lipid metabolism

Mature OLs are the primary synthesizers of cholesterol and lipids in the central nervous system, and abnormalities in their metabolism are major pathogenic factors in neurodegenerative diseases (Zou et al., 2026, 2023). Abnormal cholesterol metabolism is strongly associated with impaired myelin formation and stability; APOE4-related changes in OLs are modifiers of disease, not obligate initiators. As the strongest genetic risk factor for AD, the ApoE4 genotype can disrupt cholesterol transport and homeostasis within OLs, thereby inhibiting myelin synthesis (Blanchard et al., 2022). In patients, reduced expression of cholesterol synthesis genes in the brain leads to a loose myelin sheath structure (Tylek and Basta-Kaim, 2025). Concurrently, abnormal intracellular accumulation of cholesterol also inhibits their differentiation and maturation, reducing the number of functional OLs, which collectively exacerbates the demyelination process (Zhu et al., 2025).

Abnormal lipid metabolism can induce neurotoxicity. Inflammatory factors inhibit lipid synthases in OLs, alter the composition of myelin lipids, and impair their function (Rezaei et al., 2025). At the same time, dysregulation of sphingolipid metabolism leads to the accumulation of lipids such as ceramides, activates apoptotic pathways, and promotes OLs death (Li T. et al., 2025). Furthermore, abnormal lipid metabolism can promote the release of pro-inflammatory mediators, exacerbate neuroinflammation, and create a vicious cycle of “metabolic abnormalities–inflammation–myelin damage” (Zhao et al., 2025).

#### 3.4.2 Endoplasmic reticulum stress and activation of the unfolded protein response

Abnormal activation of endoplasmic reticulum (ER) stress and the unfolded protein response (UPR) in OLs is a key mechanism of cellular damage in neurodegenerative diseases. Abnormal protein aggregation can induce ER stress. In OLs, abnormal aggregation of proteins such as Aβ and tau leads to the accumulation of misfolded proteins in the ER, thereby activating the IRE1α- and ATF6-mediated UPR pathways. Endoplasmic reticulum stress leads to sustained activation of the UPR, causing functional disruption in OLs (Festa et al., 2024). Specifically, excessive activation of the IRE1α-XBP1 pathway promotes the secretion of pro-inflammatory factors, exacerbating inflammation. Conversely, abnormalities in the ATF6 pathway impair lipid synthesis and myelin repair (Touvier et al., 2025). Moderate activation of the integrated stress response (ISR) can exert a protective effect, but excessive activation leads to apoptosis, suggesting that modulating the intensity of the UPR may represent a potential therapeutic approach (Jeong et al., 2025).

#### 3.4.3 Downstream effects of abnormal activation of the complement pathway, NF-κB pathway, and others

Abnormal activation of the complement pathway can exacerbate OLs damage and myelin destruction through its downstream effects. Complement components (such as C3) expressed by OLs are activated; their product, C3a, activates pro-inflammatory signaling via receptors such as C3aR through an autocrine mechanism, thereby inhibiting myelin repair and inducing apoptosis (Huang et al., 2019). Concurrently, complement activation enhances the antigen-presenting function of OLs, upregulates MHC molecule expression, and exacerbates immune attacks.

Synergistic pathogenic mechanisms associated with metabolic and signaling pathway dysregulation primarily involve the abnormal activation of pathways such as the complement pathway and the NF-κB pathway. NF-κB activation promotes the release of pro-inflammatory factors and chemokines by OLs, exacerbating neuroinflammation, inhibiting cellular differentiation and maturation, and impeding myelin regeneration, thereby creating a vicious cycle (Schlett et al., 2023). Overexpression of the LINGO-1 pathway can inhibit their differentiation and myelination (Kalafatakis et al., 2023), while excessive activation of the STAT1 pathway under IFN-γ stimulation also suppresses lipid synthesis and exacerbates demyelination (Büschgens et al., 2025). These signaling pathways are interrelated and collectively mediate OLs dysfunction, thereby driving neurodegenerative processes.

## 4 Therapeutic strategies and research advances targeting oligodendrocyte lineage cells

Therapeutic strategies are strictly divided into those targeting OPCs and those targeting mature OLs.

### 4.1 Strategies for myelin repair and regeneration

#### 4.1.1 Applications of OPC differentiation promoters

As a population of stem cells that persist throughout life in the central nervous system, impaired differentiation of OPCs is one of the key causes of failed myelin repair; therefore, the development of OPCs differentiation promoters has become a major focus in the treatment of myelin regeneration. Clemastine is an anticholinergic drug that effectively promotes the differentiation of OPCs into mature OLs and accelerates myelin regeneration. Both animal models of AD and clinical trials have confirmed that it improves cognition and promotes myelin repair, making it an OPCs differentiation promoter with promising applications across multiple diseases (Feng et al., 2025).

Animal models have demonstrated that bexarotene promotes OPC differentiation and myelin regeneration by activating the RXR signaling pathway (Feng et al., 2025). However, its efficacy exhibits regional specificity (it is more effective for gray matter repair than white matter repair; De Meo et al., 2025) and is associated with adverse effects such as hypothyroidism (Vidal et al., 2021). Therefore, clinical application requires optimized strategies. Furthermore, by regulating lipid metabolism to improve OLs function, it provides a theoretical basis for its application in diseases such as AD (Tai et al., 2014).

#### 4.1.2 The enhancing effect of LINGO-1 inhibitors on OL and OPC myelin repair

LINGO-1 plays a key negative regulatory role in myelination and is primarily expressed in OLs and OPCs; it contributes to neurodegenerative diseases by inhibiting cellular differentiation and axonal myelination (Kalafatakis et al., 2023). Currently, inhibitors targeting LINGO-1 have become an important strategy for promoting myelin repair. LINGO-1 inhibitors (such as Opicinumab) can promote OPC differentiation and myelin regeneration, reduce demyelinating lesions, and improve neurological function (Strecker et al., 2024). A Phase II clinical trial (RENEW) demonstrated that it improves visual evoked potential parameters in patients with acute optic neuritis. Basic research continues to support its potential as a therapeutic target; for example, in AD models, it improves early myelin damage and cognitive function, suggesting that its efficacy may be related to the disease stage (Hanf et al., 2020). Studies indicate that LINGO-1 inhibitors enhance myelin repair capacity by promoting OPC differentiation, enhancing cell survival, and protecting axons (Strecker et al., 2024). Currently, research is shifting toward combination therapy strategies to enhance efficacy.

#### 4.1.3 Stem cell-derived OLs and OPCs transplantation technology

Advances in stem cell technology have opened new avenues for OL lineage replacement therapy. Transplantation of stem cell-derived OLs or OPCs can directly replenish populations of functionally normal cells and promote myelin repair. Human pluripotent stem cells (hPSCs) can be efficiently induced to differentiate into OLs and OPCs. In various models of neurological diseases, transplantation of these cells has been shown to effectively promote myelin repair and regeneration (Feng et al., 2023). For example, in demyelinating disease models, transplantation improves motor function; in spinal cord injury models, it promotes the recovery of neural function; and in AD models, it alleviates neuronal damage by repairing myelin and regulating Aβ metabolism (Christodoulou et al., 2023).

A key strategy for myelin repair and regeneration involves transplanting oligodendrocytes derived from stem cells (Yousof et al., 2026). Currently, OPCs demonstrate superior integration and myelin regeneration capabilities compared to mature cells. To address the issue of immune rejection, research efforts include differentiating autologous induced pluripotent stem cells (iPSCs) and establishing a bank of HLA-matched allogeneic stem cells (Feng et al., 2023). However, this technology still faces challenges such as complex manufacturing processes and the difficulty of scaling up production (Table 2).

**Table 2:** Summary of therapeutic strategies targeting OL lineage, development stage, efficacy evidence and limitations.

Therapeutic strategy	Representative drugs/ technologies	Development stage	Efficacy evidence	Core limitations	References
Myelin repair and regeneration	Clemastine; bexarote; LINGO-1 inhibitors (opicinumab); OPC transplantation	Preclinicl; phase II clinical	Promote OPC differentiation; accelerate myelin repair; improve cognitive/motor function	Bexarotene: regional specificit & hypothyroidism; opicinumb needs combination; immune rejection	Narine et al., 2023; Strecker et al., 2024
Inflammation and immunomodulation	C3/C5 inhibitors; TNF/IFN-γ/IL-17 antibodies	Preclinicl; phase I/II clinical	Reduce OL apoptosis; inhibit neuroinflammation; improve myelin integrity	Non-selective TNF inhibition blocks protective signals; poor BBB permeability	Huang et al., 2019; Xu et al., 2023
Correction of metabolic disorders	Cholesterol modulators; TUDCA; Metformin	Preclinicl; clinical repurposing	Correct lipid/energy metabolism; alleviate ER stress; stabilize myelin	Off-target effects; unclear long-term safety; limited human data	Chen, 2026; Narine et al., 2023; Zhu et al., 2025
Genetic and molecular targeted therapy	OL-specific BACE1 inhibitors; ASOs; targeted GDNF delivery	Preclinicl; phase I clinical	Precise Aβ regulation; reverse OL pathological phenotype; targeted neuroprotection	High cost; delivery efficiency; potential off-target	Lech et al., 2025; Pratsch et al., 2023

### 4.2 Inflammation and immunomodulatory interventions

#### 4.2.1 Development and applications of complement system inhibitors

Excessive activation of the complement system is a key mechanism underlying OLs damage and myelin destruction (Given et al., 2025). Accumulation of complement components in pathological regions has been observed in neurodegenerative diseases such as AD, and inhibitors targeting the complement system have emerged as a potential strategy for protecting OLs (Tenner and Petrisko, 2025). In inflammatory and immunomodulatory interventions, complement inhibitors primarily target C3 and C5. Studies have shown that inhibiting the C3 signaling pathway effectively reduces OLs apoptosis and demyelination while improving myelin integrity (Xu et al., 2023). In contrast, although C5 inhibitors can reduce the formation of the membrane attack complex, their role in myelin repair is relatively limited. This suggests that interventions targeting early-stage complement components (such as C3) hold greater therapeutic potential in regulating neuroinflammation and myelin damage (Garton et al., 2024).

In an EAE (experimental autoimmune encephalomyelitis) model, antibodies targeting complement factor B reduce chronic demyelination (Zhang and Li, 2025); inhibition of the complement component C1q reduces the formation of inflammatory OLs (Altunay et al., 2024), thereby alleviating neuroinflammation and myelin damage. These findings provide important evidence for the development of neuroimmune therapeutic strategies centered on complement inhibitors.

#### 4.2.2 Protective effects of inflammatory cytokine neutralization strategies on OLs

Excessive levels of inflammatory cytokines (such as TNF, IFN-γ, and IL-17) in the microenvironment of neurodegenerative diseases can severely impair the function of OLs; however, neutralizing these factors or blocking their signaling pathways can effectively protect these cells from damage (Blaszczyk et al., 2025).

TNF exerts a dual effect on OLs: it induces apoptosis via TNFR1 signaling, while promoting the differentiation of their progenitor cells (OPCs) and myelin repair via TNFR2 signaling (Fiedler et al., 2023). Therefore, non-selective inhibition of TNF may simultaneously block these protective signals. This suggests that future therapeutic strategies should focus on specific targeting, such as selectively activating TNFR2 or inhibiting TNFR1, to achieve precise protection and repair of OLs (Garton et al., 2024).

The effects of IFN-γ are concentration- and time-dependent. High concentrations of IFN-γ induce OL apoptosis and inhibit myelination, whereas low concentrations exert a protective effect (Holloway et al., 2023). In the EAE model, IFN-γ alleviates symptoms during the acute phase but impedes myelin repair during the recovery phase, suggesting that strategies to neutralize its effects must be precisely timed and dosed (Tichauer et al., 2023). Furthermore, IL-17 can inhibit the differentiation of OPCs by activating the NOTCH1 signaling pathway. However, blocking IL-17 promotes myelin regeneration (Wang et al., 2025), providing a new therapeutic target for combined immunotherapy targeting Ols (Table 2).

### 4.3 Correction of metabolic disorders

#### 4.3.1 Improvements in OLs function induced by cholesterol metabolism modulators

**Figure 3:**
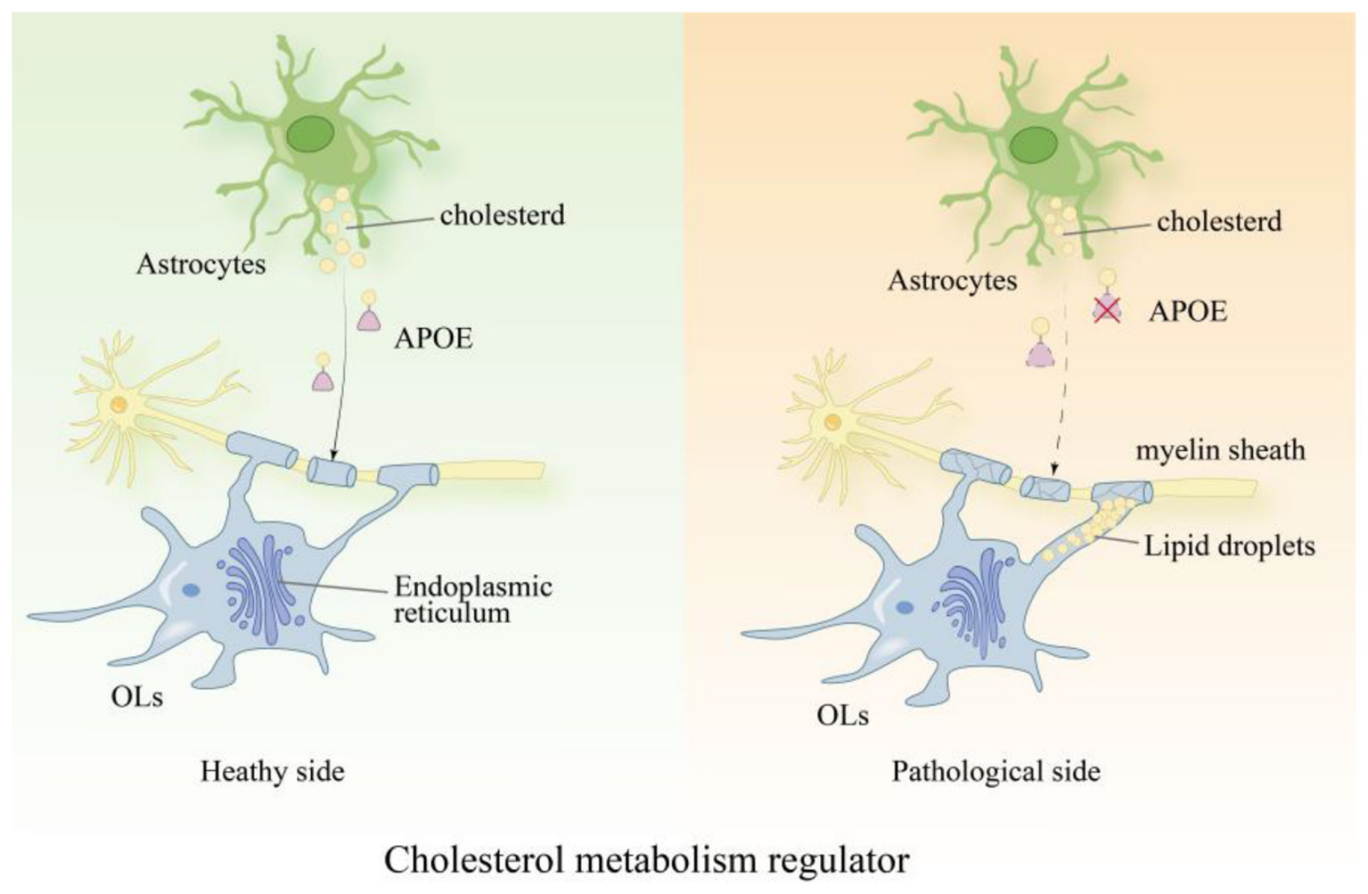
APOE4 disrupts cholesterol homeostasis in oligodendrocytes and impairs myelination: the APOE4 allele disrupts cholesterol homeostasis in OLs through two distinct pathways, leading to impaired myelination: first, APOE4 causes abnormal cholesterol accumulation within OLs, triggering endoplasmic reticulum stress and inhibiting cell differentiation and myelin synthesis; on the other hand, cholesterol derived from astrocytes cannot be effectively transported to OLs due to APOE4-mediated transport defects, resulting in a shortage of raw materials for myelin synthesis. The combined action of these two mechanisms ultimately leads to reduced myelination.

Cholesterol is a core component of myelin sheaths; disturbances in cholesterol metabolism within OLs directly lead to impaired myelin synthesis and cellular dysfunction (Zhu et al., 2025). The APOE4 allele disrupts cholesterol homeostasis in OLs, resulting in impaired myelin formation (Figure 3; Blanchard et al., 2022). Studies have shown that the use of inhibitors of the cholesterol biosynthetic pathway (such as inhibitors targeting CYP51, TM7SF2, or EBP) can promote OLs maturation and improve myelin integrity in AD models (Pleshinger et al., 2022; Sax et al., 2022). Furthermore, supplementation with cholesterol precursors or the use of cholesterol metabolism modulators can effectively improve OLs function and promote their differentiation and myelin regeneration (Fang et al., 2024). Preclinical evidence further suggests that modulating genes associated with cholesterol efflux helps reduce the accumulation of myelin debris, thereby creating a supportive environment for repair (Huynh et al., 2024).

#### 4.3.2 The neuroprotective effects of bile acid derivatives

**Figure 4:**
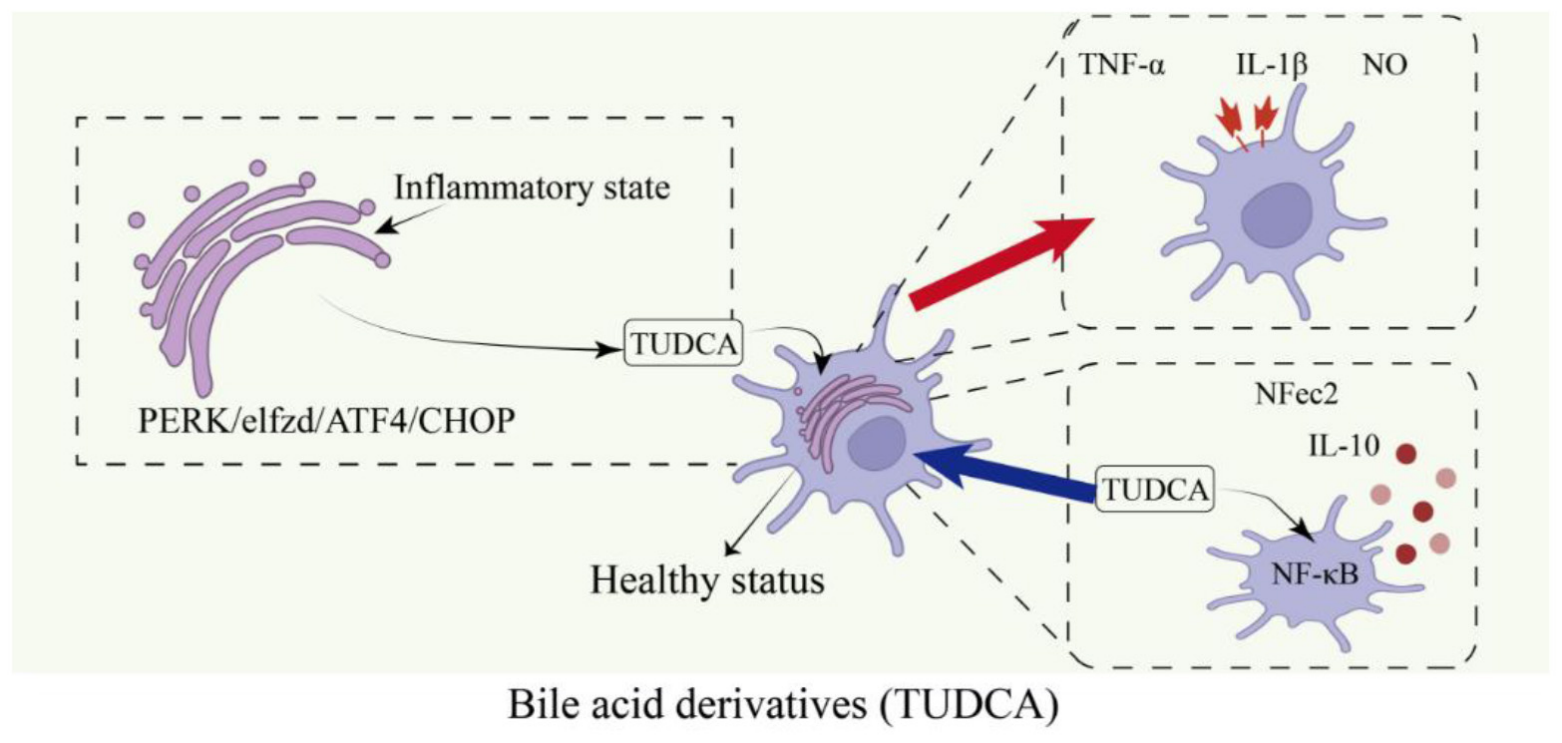
TUDCA alleviates oligodendrocyte injury and demyelination via regulating ER stress and inhibiting NF-κB pathway: TUDCA protects OLs and alleviates demyelination through a dual mechanism: on the one hand, TUDCA modulates the PERK/eIF2α/ATF4/CHOP endoplasmic reticulum stress pathway, alleviating stress within OLs and reducing apoptosis; On the other hand, TUDCA inhibits the NF-κB pathway, reducing the release of pro-inflammatory factors (such as TNF-α, IL-1β, and NO) by microglia while promoting the production of anti-inflammatory factors (such as IL-10), thereby calming the inflammatory microenvironment. The synergistic action of these two pathways effectively protects the survival and function of OLs, ultimately alleviating demyelination damage.

Bile acids and their derivatives exert a protective effect on OLs by modulating inflammatory responses, oxidative stress, and cell survival signaling; among these, tauroursodeoxycholic acid (TUDCA) has been the most extensively studied. TUDCA can alleviate OLs damage and demyelination by regulating endoplasmic reticulum stress and inhibiting the NF-κB pathway (Figure 4; Garton et al., 2024). Animal studies have demonstrated that TUDCA reduces systemic and central inflammation, promotes myelin repair, and improves neurological function (Zhan et al., 2026).

Furthermore, bile acid derivatives can enhance myelin stability by correcting cholesterol metabolism disorders in OLs and reducing Aβ-induced cytotoxicity (Figure 4; Ziar et al., 2025). Relevant studies have shown that they can also modulate the gut microbiota and reduce the proportion of pro-inflammatory immune cells throughout the body (Lin et al., 2026). In addition, some derivatives directly promote OLs survival and myelin regeneration by activating the FXR signaling pathway, highlighting their role in neuroprotection (Butcher and Arthur, 2025).

#### 4.3.3 Targeted regulation of energy metabolic pathways

OLs rely on glycolysis and oxidative phosphorylation to maintain myelin synthesis and cellular function. Under pathological conditions, their energy metabolism undergoes reprogramming, which in turn exacerbates pathological damage (Li and Sheng, 2023). Therefore, targeted regulation of energy metabolism pathways holds promise as a potential therapeutic strategy.

**Figure 5:**
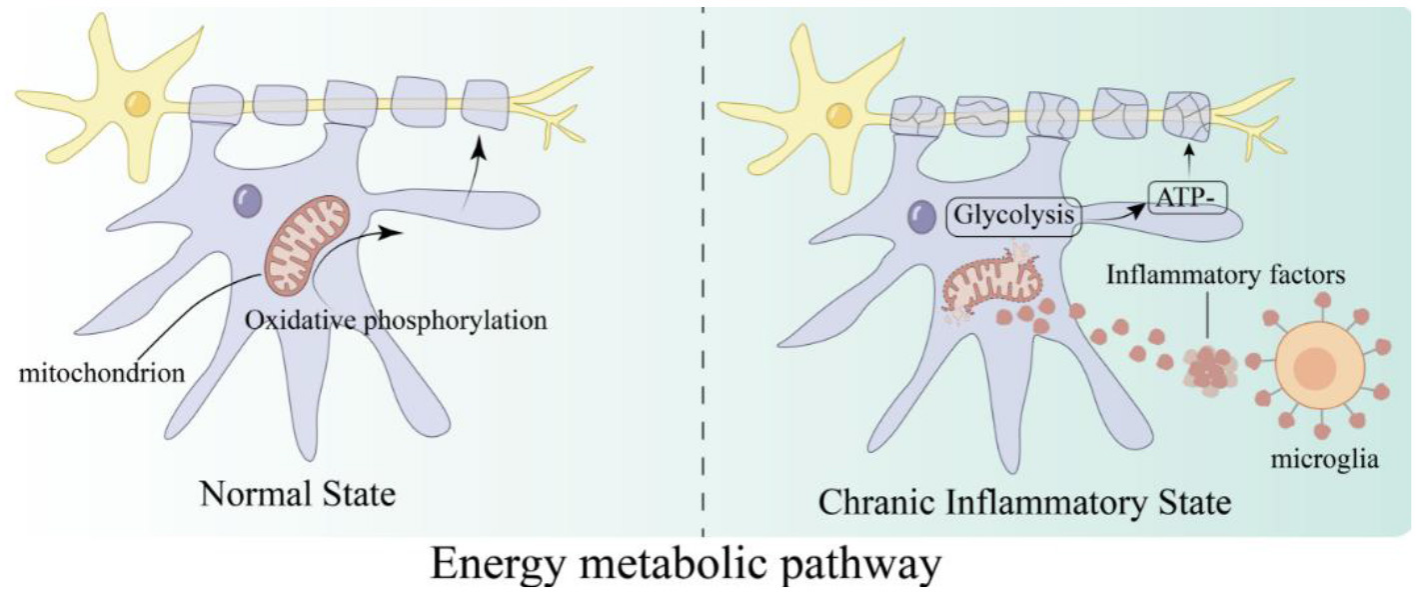
Chronic inflammation induces metabolic switch from OXPHOS to glycolysis in oligodendrocytes, leading to impaired myelin repair: chronic inflammation activates microglia to release inflammatory factors, which attack the mitochondria of OLs, inhibit efficient oxidative phosphorylation, and force OLs to switch to inefficient glycolysis. This leads to a sharp drop in ATP production, which in turn affects the energy and raw materials required for myelin synthesis, ultimately inhibiting myelin repair.

In a chronic inflammatory environment, OLs shift from oxidative phosphorylation to glycolysis, leading to insufficient energy supply and inhibiting myelin repair (Figure 5; Louie et al., 2024). The use of glycolysis inhibitors such as metformin can reverse this metabolic reprogramming (Narine et al., 2023). Concurrently, activation of the AMPK pathway enhances mitochondrial function and the efficiency of oxidative phosphorylation, thereby synergistically correcting metabolic dysregulation and promoting myelin repair (Narine et al., 2023).

In AD, Aβ deposition can impair mitochondrial function in OLs, leading to impaired oxidative phosphorylation. Studies have shown that targeted modulation of energy metabolism pathways (such as activation of PGC-1α) can improve cellular energy supply and myelin repair, and mitigate Aβ toxicity (Tang et al., 2025). Additionally, supplementation with metabolic intermediates such as pyruvate can serve as an alternative energy source, alleviating myelin damage and cognitive decline (Table 2; Chen, 2026).

### 4.4 Genetic and molecular targeted therapy

#### 4.4.1 Regulation of Aβ generation by OLs-specific BACE1 inhibition

Traditionally, neurons have been regarded as the primary source of Aβ. Recent studies have revealed that OLs also express APP, BACE1, and the γ-secretase complex, making them an important source of Aβ production and playing a critical role in AD (Sasmita et al., 2024). In APP NL-G-F AD model mice, specific inhibition of BACE1 in OLs significantly reduced the burden of Aβ plaques in the brain (by approximately 25%) and improved neuronal hyperexcitability (Ishii et al., 2024). Human iPSC-derived OLs can generate Aβ with a higher Aβ42/40 ratio *in vitro*, making them more prone to forming toxic aggregates, while BACE1 inhibitors can effectively suppress their production (Simons et al., 2024).

OLs specifically inhibit BACE1, thereby precisely regulating Aβ production and avoiding off-target effects caused by interactions with neuronal substrates (such as SEZ6; Pratsch et al., 2023), offering a new strategy for the treatment of AD.

#### 4.4.2 Reversal of disease-associated OLs characteristics using ASO technology

Antisense oligonucleotides (ASOs) can specifically bind to the mRNA of target genes to achieve post-transcriptional regulation, making them highly effective tools for reversing disease-associated phenotypes in OLs (Tan et al., 2024). In a model of spinocerebellar ataxia type 3 (SCA3), disease-associated OLs exhibit specific gene expression abnormalities. The use of ASOs targeting these abnormal genes effectively reverses their pathological features and restores myelin structure and function (Schuster et al., 2024).

In AD, genes such as LINGO1 and BIN1 are abnormally overexpressed in OLs, thereby inhibiting the myelin repair process. Studies have shown that ASOs targeting these genes can cross the blood-brain barrier and specifically reduce their expression in OLs (Kenigsbuch et al., 2022; Ziar et al., 2025), thereby promoting myelin regeneration and helping to improve inflammation-mediated damage to the neural microenvironment (Kenigsbuch et al., 2022).

#### 4.4.3 Targeted delivery of neuroprotective factors

Neuroprotective factors (such as glial cell-derived neurotrophic factor, GDNF) can mitigate pathological damage by supporting the survival of OLs and inhibiting apoptotic signaling. However, their systemic delivery faces challenges such as low blood-brain barrier permeability and off-target effects; therefore, the development of targeted delivery technologies is crucial.

GDNF protects OLs by activating the PI3K/Akt pathway and promotes myelin repair (Ziar et al., 2025). The use of a viral vector carrying an OLs-specific promoter enables precise targeted delivery of GDNF, effectively enhancing cell survival and myelin regeneration in the affected area (Temple, 2023).

In the AD model, targeted delivery of GDNF effectively regulates cholesterol metabolism in OLs and reduces Aβ production (Kawade and Yamanaka, 2023). Furthermore, stem cell-based delivery strategies—such as using genetically modified iPSC-derived OLs to continuously secrete GDNF—not only replenish cells but also provide nutritional support to surrounding neurons (Table 2; Lech et al., 2025).

## 5 Summary and outlook

### 5.1 Summary

Oligodendrocyte lineage cells (mature OLs and OPCs) are not merely supportive cells for neurons, as traditionally believed; rather, their functional abnormalities are not primary initiators in most cases, but are critical contributors and amplifiers in the progression of neurodegenerative diseases (Han et al., 2022); most evidence is associative or model-derived, with limited human causal data for disease onset. OL lineage cells exhibit disease-specific pathological phenotypes and context-dependent responses across different diseases: AD is characterized by emergent evidence of OLs-derived Aβ production, tau deposition, and disrupted cholesterol metabolism (Huang et al., 2025; Zou et al., 2026); PD is marked by GPR37-mediated activation of the inflammatory axis and transcellular transmission of α-synuclein; HD is characterized by mHTT-driven transcriptional dysregulation, impaired OPC maturation, and defective metabolic support, with tau abnormalities as secondary events (Li X. et al., 2025).

We propose a unified mechanistic model to explain the heterogeneous OL pathologies across AD/PD/HD: (1) Core trigger: AD (Aβ/tau), PD (α-syn), HD (mHTT); (2) Regulatory hub: AD (cholesterol metabolism), PD (GPR37-IL-6 inflammatory axis), HD (transcriptional inhibition); (3) Final common pathway: myelin damage + axonal metabolic failure + inflammatory amplification (Festa et al., 2024); (4) Phenotype specificity: determined by the interaction between pathogenic proteins and OL-intrinsic signaling pathways. The cross-regulation of four core mechanisms—myelin damage and axonal degeneration, immune-inflammatory imbalance, abnormal protein toxicity, and metabolic signaling disruption—forms a vicious cycle that drives the progressive development of the disease (Sasmita et al., 2024).

This review clarifies the distinctions between primary OL autonomous pathology, secondary responses to neuronal injury, and disease-associated white matter changes (Festa et al., 2024). Targeted therapies for OL lineage have evolved into four core strategies: myelin repair, immune modulation, metabolic correction, and gene targeting. While each of these strategies has demonstrated neuroprotective effects in basic research, current challenges include limited efficacy of single-target therapies, poor blood-brain barrier permeability, and significant side effects during clinical translation. Multi-target combination therapy, which can simultaneously restore core functions of OL lineage cells—including myelination, metabolic support, and immune regulation—has emerged as a key direction for clinical translation. This review systematically elucidates the multidimensional core roles of oligodendrocyte lineage cells in neurodegenerative diseases, confirming that white matter damage mediated by OLs and OPCs interacts with neuronal pathological changes to jointly constitute a comprehensive pathological network of the disease. It provides a new perspective on white matter for elucidating disease pathogenesis and lays a theoretical foundation for the development and clinical translation of novel OLs-targeted therapeutic strategies.

### 5.2 Future research directions

#### 5.2.1 Identification of OL and OPC heterogeneity and disease-specific subtypes

OLs exhibit high heterogeneity, and the formation of disease-specific subtypes in various conditions is a central focus of current research (Sadick et al., 2022). Single-cell sequencing has revealed that OLs in AD exhibit characteristics such as disrupted cholesterol metabolism and downregulation of myelin genes, and that a disease-associated subtype is present near Aβ plaques (Mathys et al., 2023). OPCs in the lesion area exhibit varying states of differentiation and inflammatory response, with some even experiencing differentiation arrest. However, the molecular definitions, functional differences, and reliable biomarkers of these disease-specific subtypes remain unclear. Future studies should integrate multi-omics and functional analyses to establish a molecular classification system, thereby elucidating the dynamic roles of each subtype in disease progression.

#### 5.2.2 Molecular mechanisms underlying crosstalk between OL lineage cells and other cell types

Abnormal crosstalk between OLs and neurons, astrocytes, and microglia is a key pathological mechanism in neurodegenerative diseases, but its molecular mechanisms remain incompletely understood. TNF-α and IFN-γ released by microglia inhibit the differentiation and maturation of OPCs by modulating the balance of TNFR1/2 receptors on their surfaces; meanwhile, CSPGs secreted by astrocytes hinder the interaction between OPCs and axons, thereby impairing myelin repair. In AD, astrocytes influence the clearance of Aβ by microglia via complement C3, while Aβ produced by OLs themselves can conversely exacerbate the inflammatory activation of astrocytes. Furthermore, impairment of the metabolic support pathways between OLs and neurons exacerbates axonal degeneration in AD and PD (Festa et al., 2024).

Future research should focus on key signaling molecules such as cytokines, complement, and lipid mediators, and systematically elucidate their specific networks of action in disease.

#### 5.2.3 Applications of novel models (organoids and gene-edited animals) in mechanistic research

Organoids and gene-edited animal models have become essential tools for studying the mechanisms underlying neurodegenerative diseases (Zheng et al., 2026). Brain organoids can replicate OLs differentiation and Aβ-mediated myelin damage (Ahuja et al., 2026), but they lack a vascular and immune microenvironment (Cerneckis et al., 2023). Gene-edited animal models (such as APP NL-G-F mice) can specifically regulate Aβ production in OLs, aiding in the elucidation of its pathogenic role, but they struggle to simulate the complex pathology of sporadic diseases. In the future, it will be necessary to combine the strengths of both approaches to construct integrated models that include vascular and immune components and can simulate the progressive development of the disease, thereby providing a superior platform for mechanistic research and drug development.

#### 5.2.4 Species differences and human validation of key mechanisms

Most core mechanistic evidence is derived from rodent models or human iPSC systems, with significant species differences in OL lineage composition, myelin structure, and lipid metabolism between rodents and humans. Many key conclusions (e.g., OL-derived Aβ production) lack rigorous causal validation in human patients. Future research should prioritize cross-species comparative studies and validate mechanistic findings using human post-mortem brain tissues, *in vivo* imaging, and liquid biopsy to improve clinical translational value.
